# The *Drosophila* TRPP Cation Channel, PKD2 and Dmel/Ced-12 Act in Genetically Distinct Pathways during Apoptotic Cell Clearance

**DOI:** 10.1371/journal.pone.0031488

**Published:** 2012-02-08

**Authors:** Emeline Van Goethem, Elizabeth A. Silva, Hui Xiao, Nathalie C. Franc

**Affiliations:** 1 Medical Research Council Cell Biology Unit, MRC Laboratory for Molecular Cell Biology and Anatomy and Developmental Biology Department, University College London, London, United Kingdom; 2 The Department of Genetics, Affiliated to the Department of Immunology and Microbial Sciences, The Scripps Research Institute, La Jolla, California, United States of America; University of Massachusetts Medical School, United States of America

## Abstract

Apoptosis, a genetically programmed cell death, allows for homeostasis and tissue remodelling during development of all multi-cellular organisms. Phagocytes swiftly recognize, engulf and digest apoptotic cells. Yet, to date the molecular mechanisms underlying this phagocytic process are still poorly understood. To delineate the molecular mechanisms of apoptotic cell clearance in *Drosophila*, we have carried out a deficiency screen and have identified three overlapping phagocytosis-defective mutants, which all delete the fly homologue of the *ced-12* gene, known as *Dmel\ced12*. As anticipated, we have found that *Dmel\ced-12* is required for apoptotic cell clearance, as for its *C. elegans* and mammalian homologues, *ced-12* and *elmo*, respectively. However, the loss of *Dmel\ced-12* did not solely account for the phenotypes of all three deficiencies, as zygotic mutations and germ line clones of *Dmel\ced-12* exhibited weaker phenotypes. Using a nearby genetically interacting deficiency, we have found that the *polycystic kidney disease 2* gene, *pkd2*, which encodes a member of the TRPP channel family, is also required for phagocytosis of apoptotic cells, thereby demonstrating a novel role for PKD2 in this process. We have also observed genetic interactions between *pkd2*, *simu*, *drpr*, *rya-r44F*, and *retinophilin* (*rtp*), also known as undertaker (*uta*), a gene encoding a MORN-repeat containing molecule, which we have recently found to be implicated in calcium homeostasis during phagocytosis. However, we have not found any genetic interaction between *Dmel\ced-12* and *simu*. Based on these genetic interactions and recent reports demonstrating a role for the mammalian *pkd-2* gene product in ER calcium release during store-operated calcium entry, we propose that PKD2 functions in the DRPR/RTP pathway to regulate calcium homeostasis during this process. Similarly to its *C. elegans* homologue, Dmel\Ced-12 appears to function in a genetically distinct pathway.

## Introduction

During development of all multi-cellular organisms, homeostasis is achieved by controlling cell proliferation and cell death. Apoptosis is a genetically programmed form of cell death that is universally found across phyla [1], [2], [3]. The orderly removal, or phagocytosis, of apoptotic cells is critical in tissue remodelling. Specialized cells or ‘professional’ phagocytes, such as mammalian macrophages and neutrophils are extremely efficient at engulfing apoptotic cells [4], [5]. However, ‘non-professional’ phagocytes may also participate in this process in various tissues [6], [7]. Clearance of apoptotic cells generally results in the activation of anti-inflammatory signals, and thus may play an important role in the resolution of inflammation in mammals. Indeed it has been proposed that failure to clear apoptotic cells contributes to autoimmune diseases [8], [9], [10], [11], and genes involved in apoptotic cell clearance have mutated splice variants in patients with systemic lupus erythematosus [12], [13].

Genetic screens in *Caenorhabditis elegans* have identified many genes that participate in the clearance of apoptotic cells by neighbouring cells [14], [15], [16]. Among them are the *C. elegans death* genes, *ced*-*1*, -*2*, -*5*, -*6*, -*7*, -*10* and -*12*, which fall into two partially redundant pathways that converge onto the activation of the Rac-like small GTPase CED-10. CED-1, a single transmembrane receptor, acts autonomously in the engulfing cells and clusters around the apoptotic corpse [17]. CED-1 clustering depends on the presence in the engulfing cell of a functional CED-7, an ATP-binding cassette (ABC) transporter also found on the apoptotic cell where it was proposed to play a role in apoptotic cell ligand exposure, such as phosphatidylserine (PS) [17], [18]. CED-6 is a phosphotyrosine binding domain (PTB) containing protein that acts downstream of CED-1, possibly by interacting with its intracellular domain and thus acting as an adaptor protein of CED-1 [19]. CED-2, -5, -10 and -12 were found to act in a parallel and yet partially redundant pathway that controls actin cytoskeleton reorganization in cell corpse engulfment and cell migration [14], [15]. CED-2 is an adaptor protein with SH2 and SH3 domains [20], while CED-5 belongs to the CED-5/DOCK180/Myoblast city (CDM) family of molecule [21], and CED-12 is a pleckstrin homology (PH) domain-containing protein, which can bind SH3 domains [22], [23], [24]. CED-12 binds to CED-5, which itself physically interacts with CED-2; CED-12 then acts as a bipartite unconventional guanine exchange factor for CED-10, as demonstrated for their mammalian homologues, thus highlighting the evolutionary conservation in the molecular mechanisms of this process (reviewed in [14], [15]).

In *Drosophila*, three cell types have been reported to clear apoptotic cells: macrophages (also called plasmatocytes), glial cells and epithelial cells [25], [26], [27]. We previously reported a role for Croquemort (CRQ), a macrophage receptor related to mammalian CD36, also involved in apoptotic cell clearance [28], [29], thereby promoting *Drosophila* as a good model system in which to genetically dissect the evolutionary conserved molecular mechanisms of phagocytosis of apoptotic cells. Draper (DRPR), another *Drosophila* receptor with sequence homologies to the *C. elegans* scavenger receptor-related CED-1 also plays a role in phagocytosis of apoptotic cells [17], [30]. Both DRPR and the *Drosophila* homologue of CED-6 (Dmel\Ced-6), its adaptor, are also required in glial cells for axon pruning and the engulfment of degenerating neurons [30], [31], [32]. A Src tyrosine kinase, Src42A, which phosphorylates DRPR and another soluble tyrosine kinase of the Syk family, Shark, which binds to DRPR, were found to be essential for the signalling events downstream of DRPR [33]. Thus far, however, our understanding of the molecular mechanisms underlying phagocytosis of apoptotic cells in this model system has remained sparse.

In search of new genes required for phagocytosis of apoptotic corpses, we have carried out a genetic screen for new *Drosophila* loss-of-function (LOF) mutants where phagocytosis of apoptotic cells by embryonic macrophages is impaired [34]. We reported a role for Pallbearer (PALL), an F-box protein acting in an E3 ubiquitin ligase complex together with SKPA, a *Drosophila* Skp1 homologue; LIN19, a Cullin; and Effete (EFF), an E2 ubiquitin-conjugating enzyme, in promoting phagocytosis, thus highlighting a novel role for protein degradation via the proteasomal pathway in this process [34]. We recently found that Retinophilin (RTP), also known as Undertaker (UTA), a membrane occupation recognition nexus (MORN) repeat-containing molecule also promotes efficient phagocytosis by regulating Ca^2+^ homeostasis [35]. We further demonstrated a role for proteins controlling both endoplasmic reticulum (ER) Ca^2+^-release, such as the Ryanodine Receptor, Rya-r44F, and store-operated Ca^2+^ entry (SOCE), such as dSTIM and dOrai, in phagocytosis of apoptotic cells and bacteria, thus demonstrating a general role for Ca^2+^ homeostasis in phagocytosis [35]. Importantly, we found a genetic link between the genes encoding these proteins and both *Dmel\ced-6* and *drpr*, thus highlighting a role for the DRPR pathway in Ca^2+^ homeostasis during phagocytosis [35].

While *Drosophila* homologues of CED-2, -5, -10, and -12 exist that include CG1587, Myoblast City (MBC), Rac-2 and Dmel\Ced-12, respectively, their putative role in apoptotic cell clearance has not yet been studied in this model system. Here, we report the phenotypic characterization of three overlapping deficiencies of the 33C-E region of the genome that delete the *Drosophila* homologue of the *ced-12* gene (*Dmel\ced-12*). Although we found a role for *Dmel\ced-12* in phagocytosis of apoptotic cells by embryonic macrophages, the deletion of Dmel\*ced-12* itself could not account for the phagocytosis-defective phenotype of the deficiencies, as zygotic null mutants of *Dmel\ced-12* did not have as strong a phenotype as that of the deficient mutants. This led us to uncover the existence of at least one other gene within this deficiency region that is required for efficient phagocytosis, which we have identified as the Polycystic Kidney Disease 2 TRPP-like cation channel-encoding gene, *pkd2*. We have found a genetic interaction between a null allele of *pkd2* and at least one other neighbouring deficiency that also had a strong phagocytosis-defect phenotype. This neighbouring deficiency deletes *simu*, an EMILIN-like domain containing receptor with homology to CED-1 and DRPR that acts upstream of DRPR in glial cells and binds apoptotic cells [36], and we have further found a genetic interaction between the *pkd2* and *simu* null alleles, arguing that they act in the same pathway. We have also found genetic interactions between the *pkd2* null allele and the *rtp* deficiency, as well as *drpr* null and *rya-r44F* hypomorphic alleles. However, we have found no genetic interaction between a deficiency that deleted *Dmel\ced-12* (but not *pkd2*) and the *simu* deficiency, demonstrating that, as for its *C. elegans* and mammalian homologues, *Dmel\ced-12* is acting in a genetically distinct pathway to that of the DRPR (CED-1) pathway. Thus together our results further support Ca^2+^ homeostasis as being an integral part of apoptotic cell clearance, and demonstrate that like *simu*, *rtp* and *rya-r44F*, *pkd2* belongs to the DRPR pathway and plays a role in Ca^2+^ homeostasis during phagocytosis.

## Results

### Characterization of overlapping deficiencies of the 33B-E genomic region with phagocytosis defects

**Figure 1:**
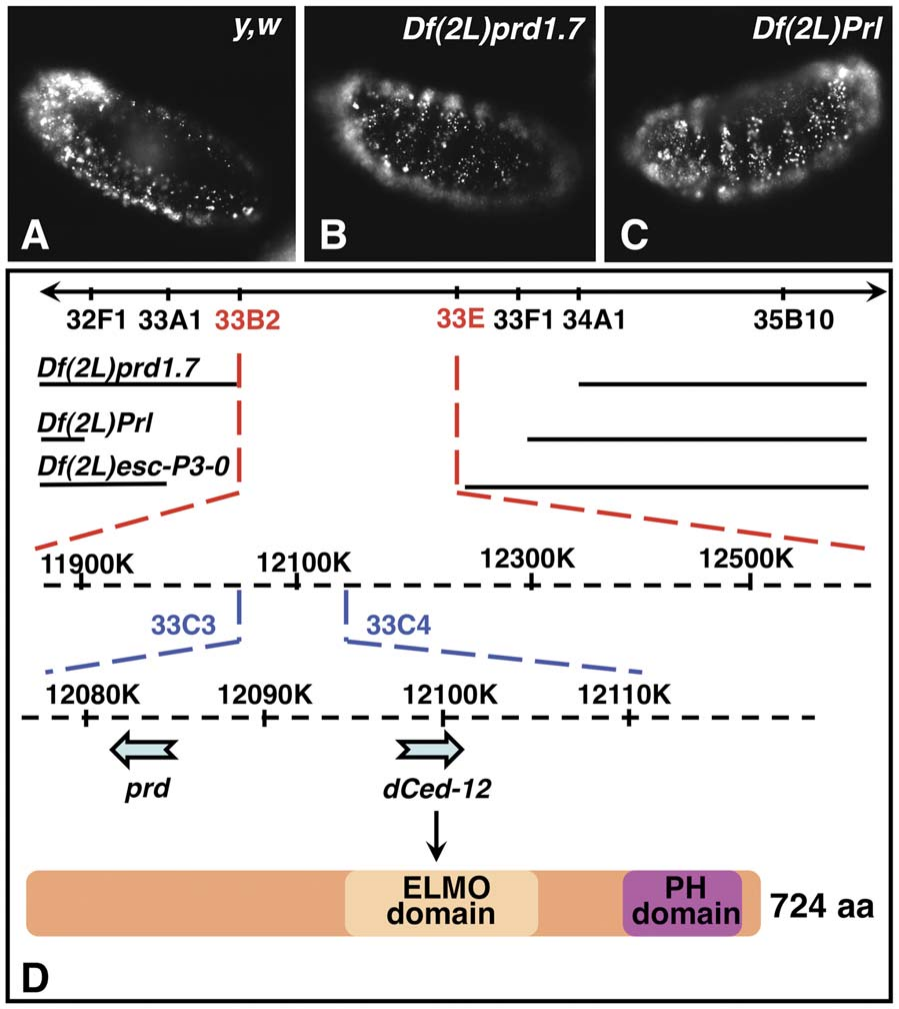
Overlapping deficiencies of the 33B-E genomic region lack clustering of AO-stained apoptotic cells. In **A–C**, embryos were aged to stage 13, stained with AO, viewed and imaged with a 20× objective under an epifluorescent Nikon microscope using the red channel, and are presented in a lateral view. **A** shows a wild-type embryo with a typical pattern of distribution of AO-stained apoptotic cells clusters around the brain lobes and in the posterior end of the embryo. **B** and **C** are *Df(2L)prd1.7* and *Df(2L)Prl* homozygous deficient embryos showing increased programmed cell death in a ‘zebra-like’ pattern along the segments of the embryo. **D** is a schematic representation of the 33B-E region of the genome with the breakpoints of all overlapping deficiencies of interest that delete both *prd* and *dced-12*. A diagram of the Dmel\ced-12 protein, with its ELMO and PH domains is also shown.

Acridine orange (AO) stains all apoptotic cells in the *Drosophila* embryo, including those already engulfed by macrophages [37]. When several apoptotic corpses are being engulfed per single macrophage, AO-staining appears in clusters, the distribution pattern of which reflects that of macrophages that have migrated throughout the embryo (**figure 1A**). We screened the deficiency kit from the Bloomington *Drosophila* stock center for deletion mutants that lacked AO-stained apoptotic cell-clusters, where macrophages may be phagocytosis-defective *in vivo* (**NCF and K. White, unpublished data**). We identified *Df(2L)prd1.7, Df(2L)Prl, and Df(2L)esc-P3-0*, three large deletions of the 33B-E region of the genome (**figure 1D**), where AO-stained apoptotic corpses mostly failed to cluster in these homozygous embryos (**figure 1B and C** **and data not shown, respectively**), arguing that they may represent novel mutants with defects in phagocytosis of apoptotic cells. In these deficient embryos, apoptotic cells were present at higher levels than in wild-type embryos, and distributed in a ‘zebra-like’ pattern along the segments of the embryo (**compare** **figures 1B and C** **with wild-type in** **figure 1A**). This apoptosis phenotype coincides with previously reported segmentation defects in these deficiency lines, as homozygous mutant embryos lack 6 out of the 12 segments normally found in wild-type embryos. Indeed, all three deficiencies delete *paired* (*prd*), a gene encoding a homeodomain transcription factor of the pair-rule family, which is required for segmentation [38]. With higher levels of apoptosis, we would have expected to observe larger clusters of AO-stained apoptotic cells. Although some large clusters were observed in the heads of these homozygous mutant embryos, we suspected that these zebra-patterns represented cells that are dying *in situ* without being engulfed by migrating macrophages, resulting from less efficient phagocytosis in these embryos.

The deficiency lines span over 600 kb and delete about 70 genes, including *Dmel\ced-12* or *Dmel\elmo*, the *Drosophila* homologue of the *C. elegans death gene ced-12*, and mammalian counterparts *elmo-1 and 2* (**figure 1D**). Similarly to *ced-12* and *elmo-1* and *2*, *Dmel\ced-12* encodes a protein that contains an ELMO domain (aa 310–490) and a pleckstrin homology (PH) domain (aa 593–673) (**figure 1D**). Dmel\Ced-12 shares 64, 81 and 79% of homology with CED-12, ELMO-1 and 2, respectively. CED-12 and ELMO-1 have been shown to be involved in the rearrangement of actin cytoskeleton in apoptotic cell clearance and cell migration [22], [23], [24]. Thus, *Dmel\ced-12* is a likely candidate gene to be responsible for the phagocytosis-defect phenotypes observed in all three deficiencies.

### Characterization of the phagocytosis phenotypes of candidate deficiencies in the 33B-E genomic region

**Figure 2:**
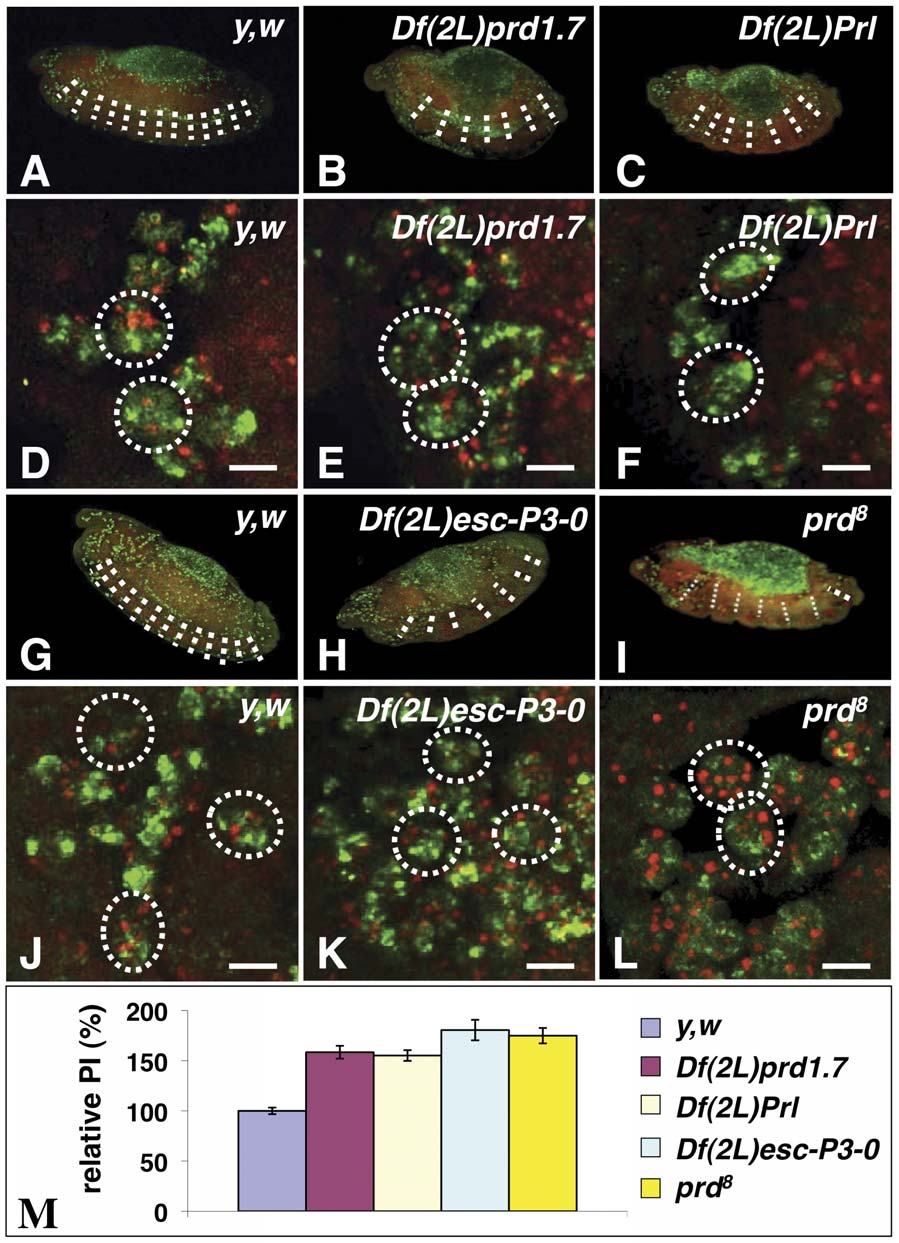
Phenotypic characterization of *all three deficiencies* and the *prd[8]* null-allele. In **A–L** embryos were aged to stage 13, fixed, their macrophages immunostained with the CRQ Ab (green) and apoptotic corpses detected with 7-AAD staining (red). Confocal images of twelve focal plans taken through macrophages were merged and projected. **A–C** are 20× lateral views of a wild-type embryo (**A**), *Df(2L)prd1.7* (**B**) and *Df(2L)Prl* (**C**) homozygous mutant embryos. Homozygous embryos of both *Df(2L)prd1.7* (**B**) and *Df(2L)Prl* (**C**) deficiencies show only 6 out of the 12 segments normally observed in wild-type embryos (**A**), as highlighted by dotted white lines. **D–F** are respective magnified views of macrophages found in the head regions of these embryos, In both homozygous *Df(2L)prd1.7* (**E**) and *Df(2L)Prl* (**F**) mutants, macrophages are able of efficiently engulf multiple apoptotic corpses. Scale bars in panels **D–F** are 5 µm. G–**I** are 20× lateral views of a wild-type embryo (**G**), *Df(2L)Esc-P3-0* (**H**) and *prd[8]* (**I**) homozygous mutant embryos. As expected the deletion of the *paired* gene causes a segmentation defect in both *Df(2L)Esc-P3-0* and *prd*-null mutant embryos. As seen in *Df(2L)prd1.7* (**B**) and *Df(2L)Prl* (**C**), *Df(2L)Esc-P3-0* (**H**) and *prd[8]* (**I**) homozygous mutant embryos also have only 6 out of the 12 segments normally observed in wild-type embryos (**A** and **G**) due to their loss of *prd* function. **J–L** are higher magnification views of macrophages within the head of *Df(2L)Esc-P3-0* and *prd[8]* homozygous mutant embryos, respectively. Mutant macrophages in both *Df(2L)Esc-P3-0* (**K**) and *prd*-null (**L**) homozygous embryos efficiently engulf multiple apoptotic corpses, with *prd*-null macrophages occasionally engulfing up to 12 corpses. Scale bars in panels **J–L** are 5 µm. Phagocytic indices for wild-type, the deficiencies, and the *prd*-null mutant embryos have been quantified and are summarised in a graph in **M**. PIs calculated from three image-stacks taken from 5 to 15 embryos of each genotype were normalized against wild type (with wild-type relative PI set as 100%) and are presented as relative PIs ± standard error of the mean (SEM) for each genotype. In D–F, and J–L, dotted white circles are indicative of individual macrophage cell bodies based on 7-AAD staining of their regular nuclei and CRQ staining.

To further characterize the phenotype of the deficiencies, we stained whole-mount homozygous deficient-embryos with the CRQ antibody (CRQ Ab), which labels all embryonic macrophages, and 7-amino actinomycin-D (7-AAD), which brightly stains apoptotic nuclei [34]. 7-AAD also stains the nuclei of all cells of the embryo, including that of macrophages, thus allowing us to monitor their ability to engulf apoptotic cells by confocal microscopy by counting the number of macrophages, as well as the number of apoptotic cells they engulf to establish their phagocytic index (PI) (i.e. the mean number of apoptotic cells per macrophage)[34]. As expected, we observed that homozygous embryos for both *Df(2L)prd1.7* and *Df(2L)Prl* deficiencies had segmentation defects that resulted in elevated levels of apoptosis in the region of the missing segments (**compare** **figures 2B and C** **with wild-type in** **figure 2A**). The CRQ immunostaining confirmed the presence of macrophages that migrated properly around the brain lobes in the head of wild-type (**supplementary figures S1A and S1D**), as well as those of *Df(2L)prd1.7* and *Df(2L)Prl* homozygous embryos (**supplementary figures S1B and S1C, respectively**). In wild-type embryos, individual macrophages were able to engulf up to 4 corpses (**figure 2D**), with a mean number of apoptotic cells per macrophage, or phagocytic index (PI) of 1.89±0.06 (normalized to 100%±3.3 in **figure 2M**). Surprisingly, and seemingly in contrast with our previous observation that AO-stained apoptotic cells appeared to mostly fail to cluster in these embryos, arguing that their macrophages may be phagocytosis-defective, we observed that *Df(2L)prd1.7* and *Df(2L)Prl* homozygous macrophages were able to efficiently engulf multiple corpses (**figures 2E and F** **, respectively**). When compared to wild type macrophages, for which we normalized the PI to 100%, *Df(2L)prd1.7* and *Df(2L)Prl* homozygous macrophages showed a 58±6 and 55±5% increase in their relative PIs, respectively (p values<0.0001)(**figure 2M**). As in *Df(2L)prd1.7* and *Df(2L)Prl*, homozygous embryos for *Df(2L)esc-P3-0*, a third overlapping *Dmel\ced-12* deletion, also had a severe segmentation defect associated with the loss of *prd*, with only six segments present (**figure 2H**). *Df(2L)esc-P3-0* homozygous macrophages also did not appear to show any major defect in migration (**Supplementary figure S1E**) or phagocytosis of apoptotic cells (**compare** **figure 2K** **with wild type macrophages in** **2J**), as they engulfed more apoptotic cells than wild-type macrophages with an 80.5±10% increase in PI (p value<0.0001)(**figure 2M**).

We suspected that these results might reflect the presence of elevated levels of apoptosis observed in these mutant embryos by comparison to wild types. Although these mutant macrophages may be less efficient at engulfing apoptotic cells, as suggested by the lack of AO-stained apoptotic cell-clusters in the deficient-embryos, their phagocytosis phenotype might be masked by the excess of apoptotic cells present. In order to separate the cell death defect from potential engulfment defects, we examined the phagocytic ability of macrophages within *prd^8^* LOF mutant embryos, as well as the pattern of distribution of apoptotic cells in these embryos. As expected, *prd^8^* LOF mutant embryos had only six segments due to the lack of PRD function resulting in a segmentation defect (**figure 2I**). As in all three deficient lines, *prd^8^* mutant macrophages also migrated properly in the head region (**supplementary figure S1F**) and engulfed significantly more apoptotic corpses than wild-type macrophages (**compare** **figures 2L** **with** **2J** **, and supplementary figure S2B with** **S2A**), with a 75±8% increase in their relative PI (**figure 2M**)(p<0.001). These results were in keeping with the elevated levels of apoptosis due to the *prd^8^* LOF mutation-associated segmentation defect.

**Figure 3:**
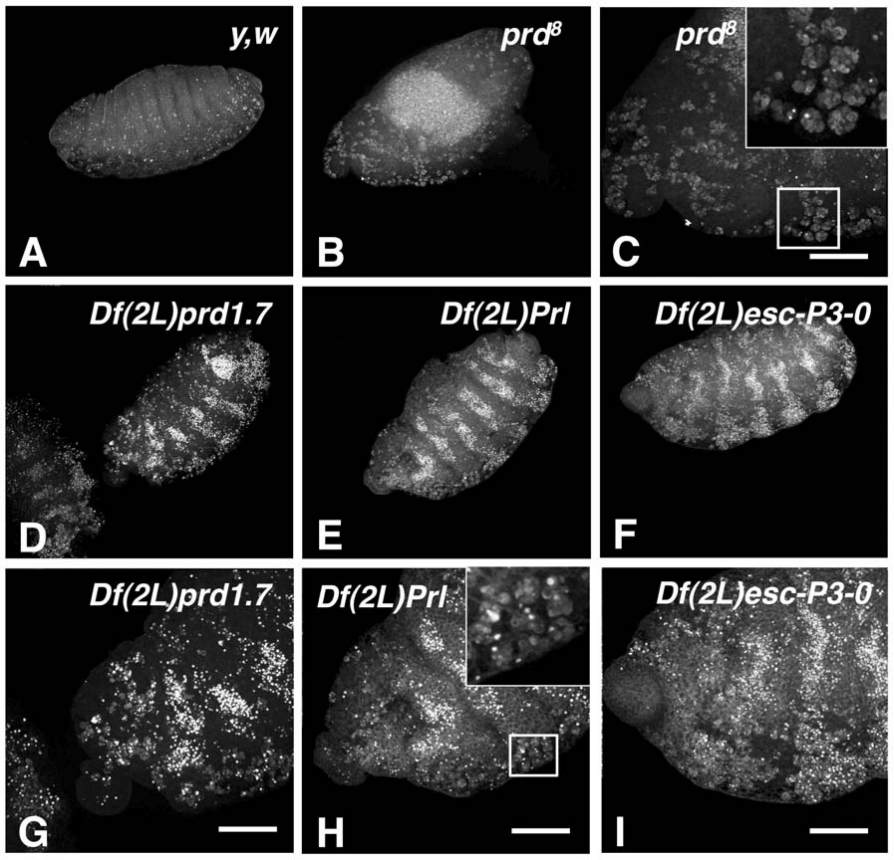
TUNEL-staining pattern argue that mutant macrophages in all three *dced-12*-deficiencies fail to efficiently clear apoptotic cells. **A–I** are projected confocal images of stage 13 embryos subjected to TUNEL. In **A** is a wild-type embryo where labelled apoptotic cells are found throughout the embryo in small clusters reflecting their engulfment by macrophages. In **B** and **C**, a 20× and enlarged views of a *prd[8]*-null mutant are shown where, as expected, TUNEL-labelled apoptotic cells are present at a higher level, with big clusters of labelled apoptotic cells seen that reflect their engulfment by *prd[8]* macrophages. In TUNEL of *Df(2L)prd1.7* (**D**), *Df(2L)Prl* (**E**) and *Df(2L)esc-P3-0* (**F**) homozygous embryos, a cell death pattern in stripes is observed along the segments, arguing that macrophages in these deficiencies are not able to clear apoptotic cells as efficiently as macrophages do in *prd[8]*-null mutants. **G–I** are 40× magnified counterparts of **D–F**, where some clusters of labelled apoptotic cells can still be observed (arrows), although the majority of labelled apoptotic cells remain scattered around the embryo. Scale bars in panels G–I are 50 µm.

Surprisingly, however, we found that the pattern of distribution of apoptotic cells in *prd^8^* mutant embryos differed from that of each of the three deficiencies, as determined by Terminal deoxyribonucleotide transferase (TdT)-mediated dUTP Nick End Labelling (TUNEL). Indeed, while all mutant embryos had increased levels of apoptosis when compared to wild-type embryos (**compare** **figures 3B and 3D–F** **with** **figure 3A**), *prd^8^* mutant embryos appeared to have fewer apoptotic cells than each of the three deficiencies. More importantly, we observed that TUNEL-stained apoptotic cells in *prd^8^* LOF were found in large clusters (**figure 3C** **and inset**). Instead, and as seen previously in their AO-staining (**see** **figure 1**), *Df(2L)prd1.7*, *Df(2L)Prl* and *Df(2L)esc-P3-0* homozygous mutants had excessive amounts of apoptotic cells in a “zebra-like” distribution pattern (**figure 3D, E and F** **, respectively**). Thus, while some clustering of TUNEL-stained apoptotic cells could still be observed in these deficiency-mutant embryos (**see inset in** **figure 3H**), and while their PIs indicated that their macrophages were capable of engulfing multiple apoptotic cells, their patterns of distribution of AO or TUNEL stained-apoptotic cells argue that they are less efficient than *prd^8^* macrophages in clearing the vast amount of apoptotic cells generated during defective segmentation.

### *Dmel\ced-12* is required for efficient engulfment of apoptotic corpses by macrophages, but does not solely account for the phagocytosis-defect observed in *Df(2L)prd1.7* mutant embryos

**Figure 4:**
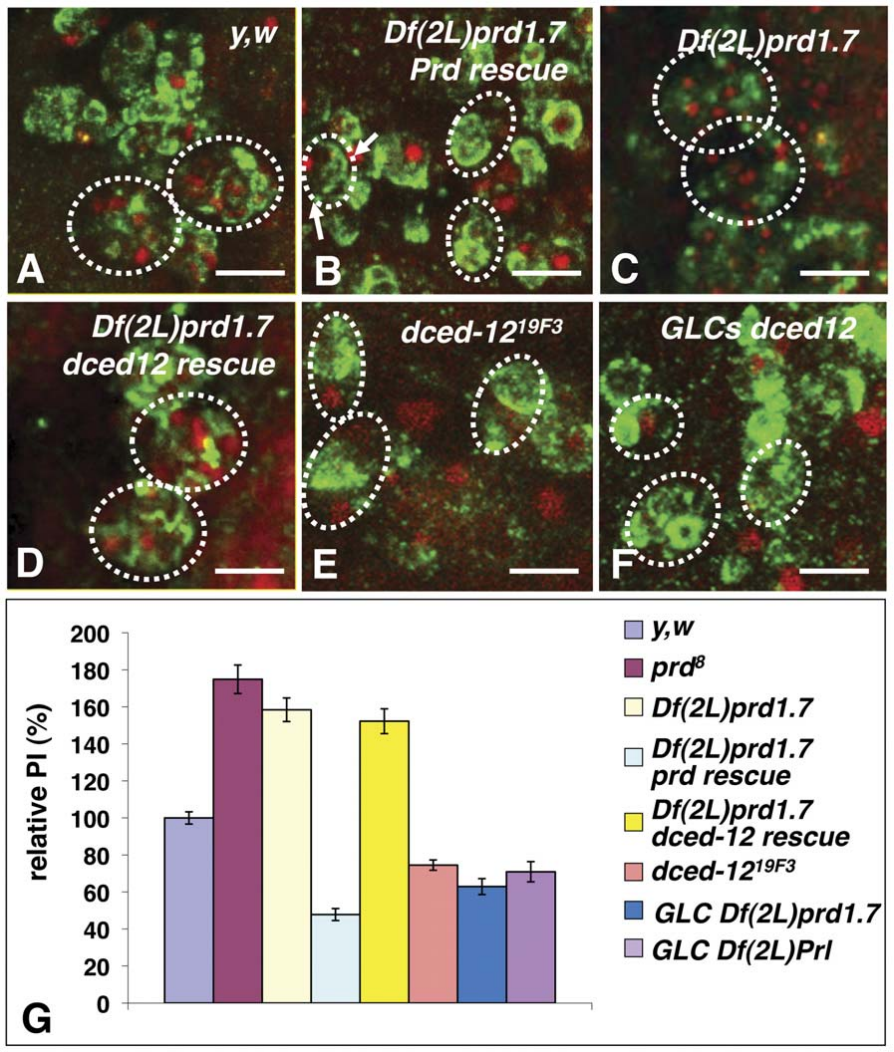
*Dmel\ced-12* is required in macrophages for efficient clearance of apoptotic cells. In **A–F** are projected confocal images of macrophages of stage 13 embryos stained with the anti-CRQ Ab (green) and where apoptotic corpses are stained with 7-AAD (red). In **A**, wild-type macrophages are shown that appear large as they engulf several apoptotic cells each. In **B**, *prd*-rescued *Df(2L)prd1.*7 homozygous mutant macrophages fail to efficiently engulf multiple corpses and appear small. *Df(2L)prd1.7* homozygous mutant macrophages where a *UAS-Dmel\ced-12*transgene is being specifically over-expressed using a *crq-gal4* driver on the third chromosome (**D**) do not engulf significantly more efficiently than in absence of the *UAS-Dmel\ced-12*transgene (**C**). In E and F are images of macrophages within the head of *dced-12[19F3]* homozygous mutant embryos (**D**) and that of macrophages of *dced-12[PB] GLCs in trans- with Df(2L)prd1.7* (**F**). Macrophages in *dced-12[19F3]* and GLCs mutant embryos can still engulf apoptotic cells, albeit at a lower efficiency than that of wild-types (**A**). Scale bars in **A–F** are 5 µm. A graph in **G** summarizes the quantification of the PIs of wild-type (yw), *Df(2L)prd1.*7, *prd*-rescued *Df(2L)prd1.*7, *dced-12*-rescued *Df(2L)prd1.*7 on the third chromosome, *dced-12[19F3]*, and *Dmel\ced-12GLCs* homozygous macrophages. Each bar represents the mean value ± SEM of the relative PIs for each genotype. Scale bars in panels **A–F** are 5 µm. In A–F, dotted white circles are indicative of individual macrophage cell bodies based on 7-AAD staining of their regular nuclei and CRQ staining.

To assess the efficiency of *Df(2L)prd1.7* deficient macrophages in the presence of wild-type levels of apoptosis, we rescued the apoptotic cell-death associated with the *prd* deletion in the *Df(2L)prd1.7* background using a previously characterized *prd* genomic rescue transgene [38]. These *prd*-rescued *Df(2L)prd1.7* macrophages were less efficient in clearing apoptotic cells than that of wild type embryos (**compare** **figure 4B** **with wild-type in** **figure 4A**) with a 52% decrease in their PI (p<0.001)(**figure 4G**). As a consequence of their failure to efficiently engulf apoptotic cells, *prd*-rescued *Df(2L)prd1.7* mutant macrophages appeared smaller in size than wild-type macrophages (**compare** **figure 4B** **with** **figure 4A**), as previously described in other phagocytosis-defective mutant macrophages, such as those of *crq*-deficient and *pallbearer* (*pall*) LOF mutants [29], [34]. Thus, these results confirm that *Df(2L)prd1.7* deletes at least one gene required for efficient phagocytosis of apoptotic corpses.

To address whether the phagocytosis defect might be associated with the LOF of *Dmel\ced-12*, we carried out a *Dmel\ced-12* rescue experiment. We generated a *UAS-Dmel\ced-12* transgenic line on the X chromosome and established a fly line carrying both *Df(2L)prd1.7* and the *UAS-Dmel\ced-12* transgenes. A *Df(2L)prd1.7* deficiency line was also established that carried a *crq-gal4* transgene on the third chromosome (a kind gift of Drs N. Perrimon and H. Agaisse). By crossing the deficiency line carrying the *UAS-Dmel\ced-12* transgene to the deficiency line carrying the *crq-gal4* driver, we were able to specifically drive the expression of *Dmel\ced-12* in *Df(2L)prd1.7* homozygous macrophages. We found that re-expressing *Dmel\ced-12* was not sufficient to significantly increase the ability of *Df(2L)prd1.7* mutant macrophages to clear apoptotic cells (**compare** **figures 4C and D**), as their PI was strikingly similar to that of *Df(2L)prd1.7* homozygous macrophages alone (relative PI = 152.2±6.7% versus 158.4±6.4%, p>0.05)(**figure 4G**). Thus, re-expressing *Dmel\ced-12* does not appear to significantly increase the ability of *Df(2L)prd1.7* macrophages to efficiently engulf apoptotic cells, arguing against the possibility that *Dmel\ced-12* is the only gene responsible for this deficiency phenotype.

As demonstrated previously, a possibility remained that the excessive number of apoptotic cells present in *Df(2L)prd1.7* embryos and the consequently elevated PI of *Df(2L)prd1.7* macrophages might mask the effect of a *Dmel\ced-12* rescue. We could not establish the necessary lines to carry a double-rescue experiment of *Df(2L)prd1.7* mutant embryos with both *prd* and *Dmel\ced-12* transgenes; therefore we assessed the phagocytosis phenotype of homozygous embryos carrying a single *Dmel\ced-12* null allele, *ced-12^19F3^* [39](**figure 4E**). We found that *ced-12^19F3^* mutant macrophages were significantly less efficient than wild-type macrophages in clearing apoptotic cells with a 25.5±2.8% decrease in their PIs (PI = 74.5%±2.8%, and p<0.001)(**figure 4G**). The PI of *ced-12^19F3^* macrophages, however, was significantly higher than that of *prd*-rescued *Df(2L)prd1.7* macrophages, which had a PI of 47.8%±3.2% (p<0.001)(**figure 4G**), arguing that *Dmel\ced-12* may not be the sole gene responsible for the phenotype seen in the deficiency embryos.

We, and others, have observed a strong maternal contribution of *Dmel\ced-12* mRNA in early embryos that may compensate for the zygotic LOF phenotype of *Dmel\ced-12* (**data not shown** and [39], [40]). Therefore we also analyzed *Dmel\ced-12* germ line clones (GLCs). We were not able to recover any GLCs with the *ced-12^19F3^* null allele in combination with either *Dmel\ced-12* deficiency. Thus, GLCs were generated with a hypomorphic allele of *Dmel\ced-12*, *ced-12^PB06760^*, in combination with either *Df(2L)prd1.7* (**figure 4G**) or *Df(2L)prl* (**figures 4F and G**). In these embryos, the maternal germ line genotype is homozygous for *ced-12^PB06760^*, and their zygotic genotype is heterozygous for *ced-12^PB06760^* and either *Df(2L)prd1.7*, or *Df(2L)prl*. We examined their phagocytosis phenotypes and observed that macrophages in these mutant embryos had a similar phenotype to that of *ced-12^19F3^* zygotic null mutant macrophages with relative PIs of 62.9±4.3%, and 70.9±5.5, respectively, versus 74.5±2.8 (with p values>0.05)(**figure 4G**). Thus, in a similar wild-type context of apoptosis, the PIs of *ced-12^19F3^* and *ced-12^PB06760^* GLCs macrophages were lower than that of wild-type macrophages (p<0.001), arguing in favour of a role for *Dmel\ced-12* in phagocytosis of apoptotic cells. However, our observation that the PIs of *dced-12^19F3^* homozygous mutant macrophages and of *Dmel\ced-12* GLCs were significantly higher than that of *prd*-rescued *Df(2L)prd1.7* macrophages, demonstrated that *Dmel\ced-12* was only partially responsible for the phenotype observed in *prd*-rescued *Df(2L)prd1.7* mutant embryos (p<0.05)(**figure 4G**). Altogether these results show that, as for *elmo* in mammals and *ced-12* in *C. elegans*, *Dmel\ced-12* plays a role in phagocytosis of apoptotic cells in the fly; these data also argue in favour of the presence of at least one other gene in the *Df(2L)prd1.7* deficiency that is required for phagocytosis. We will hereafter refer to this gene as *gene X*.

### Genetic interactions with *Df(2L)b87e25* reveal the presence of at least one novel gene required for phagocytosis of apoptotic cells in the 33E-F1 genomic region

**Figure 5:**
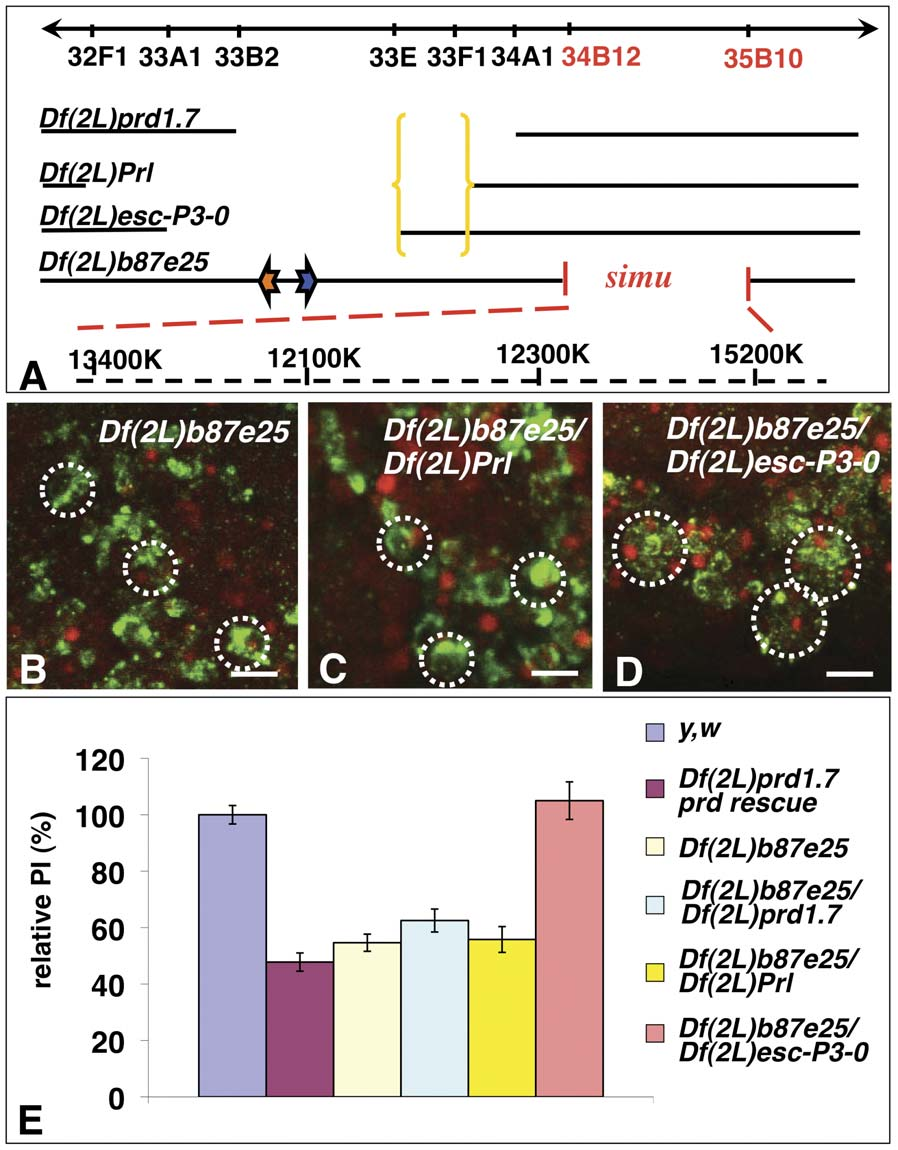
Genetic interactions with an adjacent deficiency of the 33C-E region reveals the existence of at least one novel gene required for phagocytosis of apoptotic cells in the 33E-F1 region. **A** is a schematic representation of the 33B-E region of the genome with the breakpoints of all overlapping deficiencies of interest that delete both *prd* and *dced-12*, and *Df(2L)b87e25*, a neighbouring deficiency. The *prd* gene is represented as an orange arrow, and *Dmel/ced-12* as a blue arrow. In **B–D** are projected confocal images of macrophages in stage 13 *Df(2L)b87e25* homozygous deficient embryos (**B**) and *trans*-heterozygous *Df(2L)b87e25*/*Df(2L)prd1.7*(**C**) and *Df(2L)b87e25/Df(2L)esc-P3-0* (**D**) that were stained with the CRQ Ab (green) and 7-AAD (red). Macrophages of both *Df(2L)b87e25* homozygous and *Df(2L)b87e25/Df(2L)Prl trans-*heterozygous embryos are smaller in size and generally less efficient at engulfing apoptotic cells than that of *Df(2L)b87e25/Df(2L)esc-P3-0* that appear larger with several apoptotic corpses within them. These results demonstrate the presence of at least one gene in the 33E-F1 genomic region that is required for phagocytosis of apoptotic cells (see yellow brackets in **A**). In **E** is a graph summarizing the PIs of wild-type (*y,w*), *prd*-rescued *Df(2L)prd1.*7, *Df(2L)b87e25*, homozygous macrophages and *Df(2L)b87e25/Df(2L)prd1.7*, *Df(2L)b87e25/Df(2L)Prl*, *Df(2L)b87e25/Df(2L)esc-P3-0*. Each bar represents the mean value ± SEM of the relative PIs for each genotype. Scale bars in panels **B–D** are 5 µm. In B–D, dotted white circles are indicative of individual macrophage cell bodies based on 7-AAD staining of their regular nuclei and CRQ staining.

In order to narrow down the region where *gene X* may be positioned, we screened additional overlapping and nearby deletions of the 33–34 region of the genome (**figure 5A**). We found that *Df(2L)b87e25* homozygous embryos also had phagocytosis-defective macrophages similar to that of *prd*-rescued *Df(2L)prd1.7* embryos, with a relative PI of 54.6±3.0% versus 47.8±3.2%, respectively (**figure 5B, and 5E**)(p>0.05). Single embryo mapping of the breakpoints of this novel phagocytosis-defective deficiency mutant confirmed that it deleted the 34B12-35B10 region of the genome, thus revealing the presence of yet another gene of interest in this region that is required for phagocytosis of apoptotic cells (**figure 5A**). Interestingly, a gene required for phagocytosis of apoptotic cells by glial cells, *six microns under* (*simu*) was characterized by Dr. E. Kurant and colleagues that is deleted in the *Df(2L)b87e25* mutant line [36] and is most likely responsible for the defect we observed in these *Df(2L)b87e25* homozygous embryos.

This 34B12-35B10 genomic region, however, does not overlap with either region of the previous three deficiencies. Thus, to test for any potential genetic interactions between these lines, we next assessed the phagocytosis-defective phenotypes in heterozygous mutant combinations between *Df(2L)b87e25* and *Df(2L)prd1.7, Df(2L)Prl or Df(2L)esc-P3-0*. All heterozygous mutant combinations were wild type for segmentation and consequently showed wild-type levels of apoptosis. Thus their PIs could be compared to that of *prd*-rescued *Df(2L)prd1.7* macrophages. We found that heterozygous macrophages for *Df(2L)b87e25* and either *Df(2L)prd1.7* or *Df(2L)Prl* were phagocytosis-defective (**data not shown and** **figure 5C** **, respectively**), and had relative PIs of 62.5±6.6% and 55.8±4.6%, respectively, which were similar to that of *prd*-rescued *Df(2L)prd1.7* macrophages with a relative PI of 47.8±3.2% (**figure 5E**)(with p values>0.05). Embryos heterozygous for *Df(2L)b87e25* and *Df(2L)esc-P3-0*, however, appeared wild-type for apoptosis and phagocytosis with a PI of 105±6.7% (**figures 5D, and 5E**)(p>0.05 when compared to wild type, and p<0.001 when compared to *prd*-rescued *Df(2L)prd1.7*). These results demonstrate a genetic interaction between at least one gene within the deficiency region of *Df(2L)b87e25*, which is likely to be *simu*, and *gene X*. These also allow us to localize gene X between the proximal breakpoints of *Df(2L)esc-P3-0* and *Df(2L)Prl*, i.e. within the 33E to 33F1 region of the genome (**see yellow brackets in** **figure 5A**). This localization of *gene X* is in agreement with our previously calculated PIs showing that *Df(2L)esc-P3-0* macrophages engulfed slightly but significantly more apoptotic cells than *Df(2L)prl* macrophages, with a relative PI of 180.5±10.2% versus PIs of 155.2±5.3% (p<0.05), while there was no significant difference between *Df(2L)prd1.7* and *Df(2L)prl* PIs (p>0.05)(**figure 2M**). These genetic interactions refined the region associated with *gene X* to about 306 kb containing 22 predicted genes. Finally, these results also argue that there are no genetic interactions between the gene(s) responsible for the phenotype observed in *Df(2L)b87e25* homozygous mutants and *Dmel\ced-12*.

### *pkd2* is required for apoptotic cell clearance and genetically interacts with *rya-r44F*, *rtp*, *drpr* and *simu*, *but simu does not interact with Dmel\ced-12*

Among the 22 genes of the 33E-F1 region is *pkd2*, the *polycystic kidney disease 2* gene (*pkd2*), also known as *almost there* (*amo*). *pkd2* is a homologue of the mammalian *polycystin-2* gene, which encodes PC2, a Ca^2+^-activated Ca^2+^ permeable cation channel with distant homologies to TRPP family of cation channels [41]. *Drosophila pkd2* or *amo* is required for directional sperm movement and male fertility [42], [43]. In combination with a mutation of the *ryanodine receptor* gene, *rya-r44F*, which encodes a Ca^2+^ channel on the endoplasmic reticulum (ER), a mutation in *pkd2* also lowers the rate of cell body wall contraction in *Drosophila* larvae [41]. Interestingly, we recently found a role for Rya-r44F, Retinophilin (RTP) (also known as UTA), a membrane occupation nexus repeat (MORN)-containing protein, and for molecules that promote store-operated Ca^2+^ entry (SOCE) in phagocytosis of apoptotic cells and bacteria where they regulate Ca^2+^ homeostasis [35]. *pkd2* may therefore be in the same genetic pathway as *rtp* and *rya-r44F* in controlling Ca^2+^ homeostasis during phagocytosis.

**Figure 6:**
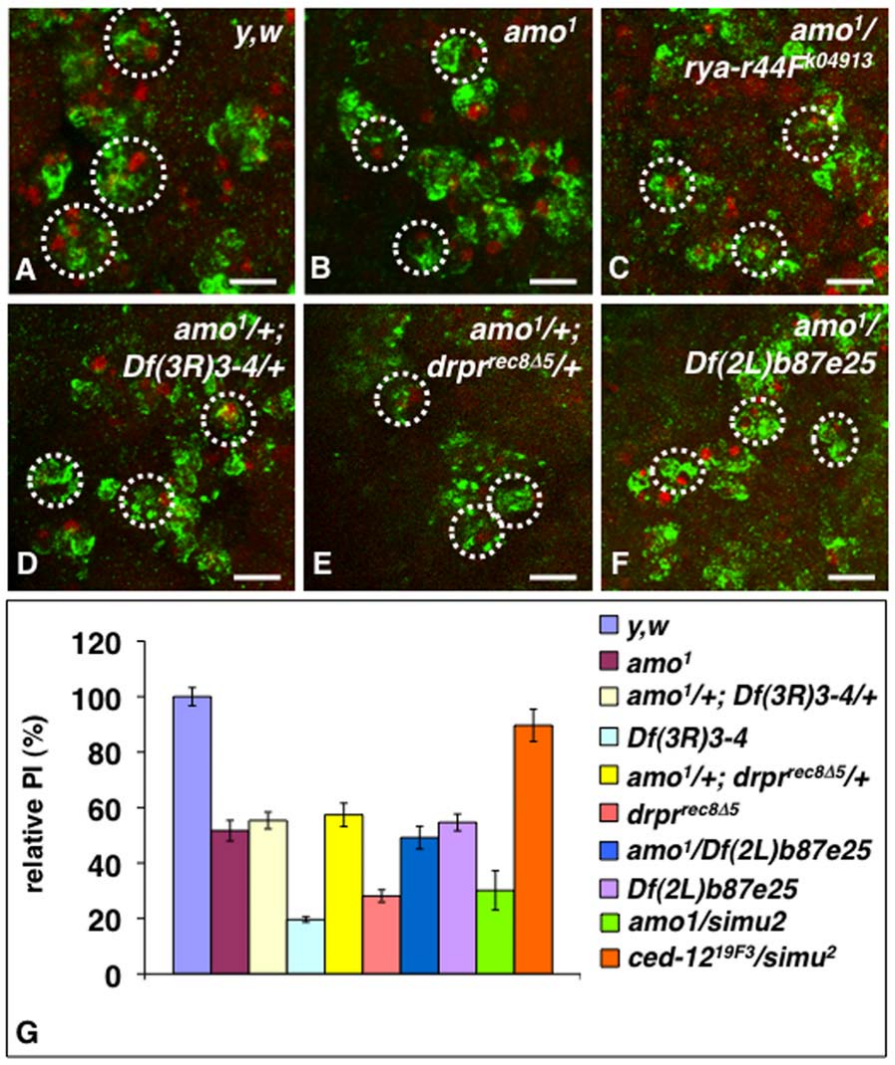
Genetic interactions between *pkd2*, *uta* and *drpr* null alleles reveal a role for *pkd2* in phagocytosis of apoptotic cells. In **A–F** are projected confocal images of macrophages in stage 13 *y,w* (wild-type) embryos (**A**), *amo^1^* homozygous embryos (**B**), *amo^1^/rya-r44F^k04913^* heterozygous embryos (**C**), double heterozygous *amo^1^/+; Df(3R)3-4/+* (**D**) and *amo^1^/+; drpr^recΔ5^/+* (**E**) embryos, and *amo^1^/Df(2L)b87e25* heterozygous embryos (**F**). All embryos were stained with the CRQ Ab (green) and 7-AAD (red). Embryos in **B–F** had macrophages that were smaller and less efficient at engulfing apoptotic cells than that of wild-type embryos (**A**). In **G** is a graph summarizing the PIs of wild-type (*y,w*), *amo1, rya-r44F^k04913^, rya-r44F^16^, Df(3R)3-, drpr^recΔ5^* and *Df(2L)b87e25* homozygous macrophages, that of *amo^1^/rya-r44F^16^*, *amo^1^/rya-r44F^K04913^*, *amo^1^/Df(2L)b87e25* and *ced-12^19F3^/simu^2^ trans*-heterozygous macrophages, as well as that of *amo^1^/+;Df(3R)3-4/+* and *amo^1^/+; drpr^recΔ5^/+* double heterozygous macrophages. Each bar represents the mean value ± SEM of the relative PIs for each genotype. Scale bars in panels **A–F** are 5 µm. In A–F, dotted white circles are indicative of individual macrophage cell body based on 7-AAD staining of their regular nuclei and CRQ staining.

We asked whether *pkd2* might be required for phagocytosis of apoptotic cells *in vivo* by assessing the phagocytosis phenotype of a previously generated knock-out allele of *pkd2, amo^1^* [43]. We found that homozygous mutant macrophages for *amo^1^* were less efficient at engulfing apoptotic cells than wild-type macrophages (**compare** **figures 6A and B**) with a relative PI of 51.7±2.6% (p<0.001)(**figure 6G**), thereby confirming a role for *pkd2* in phagocytosis of apoptotic cells. We also asked whether *pkd2*, *rya-r44F* and *rtp* might genetically interact in phagocytosis. As anticipated, we found an interaction between *pkd2* and *rya-r44F*, as heterozygous mutant macrophages for the *pkd2* knockout allele *amo^1^* and the *rya-r44F^16^* hypomorph allele were small and poorly engulfed apoptotic cells (**figures 6C**). These results are consistent with previous genetic interactions found between *pkd2* and *rya-r44F* in cell body wall contraction, and argue that *pkd2* acts in phagocytosis similarly to its role in body cell wall contraction by regulating Ca^2+^ homeostasis. We also observed a similar phenotype in double heterozygous mutant embryos for *amo^1^* and the *rtp* deficiency *Df(3R)3-4*, with smaller macrophages gathering around apoptotic corpses but engulfing poorly (**figure 6D**) with a relative PI of 55.4±2.4% similar to that of *amo^1^* homozygous macrophages (p>0.05)(**figure 6G**).

We previously linked RTP and Rya-r44F to the Dmel\Ced-6 and DRPR pathway by showing that mutations of these genes all genetically interacted *in vivo* in phagocytosis of apoptotic cells, and by further demonstrating a role for *Dmel\Ced-6* and *drpr* in SOCE in S2 cells [35]. Thus, we asked whether *pkd2* might also genetically interact with *drpr*. We found that macrophages in double heterozygous for the *pkd2* knock-out allele *amo^1^* and a null allele of *drpr*, *drpr^recΔ5^* [32], [35] poorly engulf apoptotic cells (**figure 6E**), with a relative PI of 57.4±4.2% similar to that of *amo^1^* homozygous (p>0.05)(**figure 6G**). Macrophages of single heterozygotes *amo^1^/+* or *drpr^recΔ5^/+* had relative PIs of 82.1±3.8% and 101.02±8.3%, respectively, similar to that of wild type macrophages (with p values>0.05)(**figure 6G**). Thus, these results argue that *pkd2* and *drpr* are likely to act in a same pathway.

### *simu* genetically interacts with *pkd2 but not with Dmel\ced-12*

Our earlier results demonstrating a genetic interaction between the deficiencies, *Df(2L)prd1.7 or Df(2L)Prl* and *Df(2L)b87e25*, also led us to conclude that *gene X* within the *Df(2L)prd1.7* and *Df(2L)prl* genomic regions and *simu* within the *Df(2L)b87e25* region were likely to act in the same pathway. Having identified *gene X* as *pkd2*, we re-assessed the genetic interaction between the *pkd2* knockout allele *amo^1^* and *Df(2L)b87e25*. As anticipated, macrophages in *amo^1^/Df(2L)b87e25* heterozygous embryos were small and engulfed apoptotic cells as poorly as *amo^1^* or *Df(2L)b87e25* homozygous macrophages (**figure 6F**), with a PI of 49.2±4.1% versus 51.7±2.6% and 54.6±3%, respectively (**figure 6G**)(with p values>0.05). To further test whether *pkd2* genetically interacted with *simu* in this deficiency region, we next assessed the phagocytosis phenotype of macrophages in heterozygous embryos for *amo^1^* and a null allele of *simu*, *simu^2^* (a kind gift of Dr. E. Kurant [36]). We found their phagocytic ability to be reduced to a relative PI of 30.1±7.1% (**figure 6G**), This PI is similar to that of *drpr^recΔ5^* and *Df(3R)3-4* homozygous macrophages (**figure 6G**), and consistent with previous data reporting the engulfing capacity of *simu^2^* homozygous mutants at about a third of that of wild-type macrophages [36]. This PI, however, is slightly lower than that of *amo^1^* (PI of 51.7±2.6%, p<0.05) and *Df(2L)b87e25* homozygous macrophages (PI of 54.6±3.0%, p<0.001), as well as *amo^1^*/*Df(2L)b87e25* heterozygous macrophages (PI of 49.2±4.1%, p<0.001) suggesting that the *Df(2L)b87e25* deficiency may have slightly elevated levels of apoptosis or delete a phagocytosis suppressor. We previously reported that macrophages of *Df(3R)3-4* heterozygotes were wild type for phagocytosis [35]. Here we further report that macrophages of single heterozygotes for either *amo^1^* or *simu^2^* had relative PIs of 82.1±8.3% and 95.4±3.8%, respectively, similar to that of wild type macrophages (with p values>0.05)(**figure 6G**). Thus, together these results argue that *pkd2* genetically interacts with *simu*, although its role in apoptotic cell clearance appears less prominent than that of *simu*, *drpr and rtp*. Thus, PKD2 is likely to play a role in regulating Ca^2+^ homeostasis during phagocytosis, in concert with RTP, Rya-r44F, Dmel\Ced-6 and DRPR. This is further supported by recent publications (since the first submission of this manuscript) reporting that the mammalian counterpart of PKD2, Polycystin-2 (also known as PC2, which is encoded by the *pkd2* gene) can modulate ER Ca^2+^ release and play a role in SOCE [44], [45].

Finally, our earlier results demonstrating a lack of genetic interaction between the *Df(2L)esc-P3-0* and *Df(2L)b87e25* deficiencies also argued that *Dmel\ced-12* and *simu* may not be genetically interacting and are likely to act in parallel or branched pathways. To further test this, we assessed the PI of heterozygotes macrophages for *ced-12^19F3^* and *simu^2^*. We found that both single heterogygous macrophages for *ced-12^19F3^* and *simu^2^*, and their double heterozygous macrophages all had PIs similar to that of wild type (PIs of 96.1±5.8%, 95.4±3.8% and 89.6±5.8%, respectively, with p values>0.05 when compared to wild type). Thus, like its *C. elegans* and mammalian counterparts, Dmel\Ced-12 is likely to act in a parallel pathway to the SIMU/DRPR pathway.

## Discussion

With the goal to pursue the genetic dissection of the molecular mechanisms underlying apoptotic cell clearance, we have characterized four *Drosophila* deficiency mutants with defects in this process, and have identified the homologue of *C. elegans* CED-12, Dmel\Ced-12, as being required for phagocytosis. We propose that, as with its *C. elegans* counterpart, Dmel\Ced-12 is likely to act as a Guanidine Exchange Factor (GEF) for the small GTPase RAC2, which is also required for apoptotic cell clearance in the fly (***NCF, unpublished data***), and to promote actin cytoskeleton rearrangement during phagocytosis. As in *C. elegans*, our results further argue that Dmel\Ced-12 also acts in a genetically distinct pathway to that of DRPR, the CED-1 homologue.

Our previous data demonstrated a role for RTP (UTA) in SOCE and apoptotic cell clearance, and linked RTP to the DRPR pathway [35]. Here, we have found a novel role for the TRPP-like cation channel, PKD2, in apoptotic cell clearance, and gathered evidence that it acts in the DRPR pathway, arguing that PKD2 is likely to also regulate Ca^2+^ homeostasis during phagocytosis. Calcium (Ca^2+^) was shown to play a role in phagocytosis in mammalian cells, but its precise role in this process remains unclear and somewhat controversial. A rise in intracellular Ca^2+^ concentration ([Ca^2+^]i) in mammalian phagocytes was previously described during phagocytosis of various particles, but the molecular mechanisms underlying this rise in [Ca^2+^]i are poorly understood [46], [47], [48]. Thus our results on PKD2 provide us with a new component of the molecular machinery underlying Ca^2+^ homeostasis in phagocytosis.

At least one other *Drosophila* TRP channel, TRPML, was proposed to play a role in apoptotic cell clearance by macrophages and glial cells in a model for the neurodegenerative disorder known as Mucolipidosis type IV (MLIV), a lysosomal storage disorder with severe impairment in motor neurons [49]. In mammalian macrophages, a Ca^2+^-release activated Ca^2+^ (CRAC) channel activity and more recently a TRP channel activity were also detected, yet a role for store-operated Ca^2+^ Entry (SOCE) in controlling Ca^2+^ homeostasis during phagocytosis has remained controversial [50], [51], [52]. A TRP channel of the C family, TRPC1, has now been associated with SOCE in mammalian systems [53], and thus TRP channels may be part of a large multi-molecular complex that regulate Ca^2+^ homeostasis. Although evolutionary distant from PKD2, TRPC1 has been shown to participate in SOCE by physically interacting and forming a ternary complex with the ER Ca^2+^ sensor STIM1 and the CRAC channel ORAI1 [54]. Thus PKD2 may play a role in SOCE by physically interacting with dSTIM and dOrai and future studies will be required to test this hypothesis. Of note is that PKD2 possesses an ER retention domain, arguing that PKD2 might indeed play a role at the ER level rather than at the plasma membrane, however further studies will be required to assess its subcellular localization and function in Ca^2+^ homeostasis.

Like mammalian immunoreceptors, DRPR bears an immunoreceptor tyrosine-activation motif (ITAM)[33]. During axon pruning by *Drosophila* glial cells, the ITAM of DRPR is phosphorylated by Src-tyrosine family member, Src42A, which leads to the binding onto DRPR of Shark, a non-tyrosine kinase of the Syk and Zap-70 family [33]. The activity of such kinases can be Ca^2+^-dependent in mammalian cells [55], [56], as well as promote intracellular Ca^2+^ changes [57], [58]. Thus, our working hypothesis is that an initial increase in [Ca^2+^]i either via release of Ca^2+^ from the ER and/or via SOCE activates Src42A and DRPR phosphorylation thereby promoting the recruitment of Dmel\Ced-6 and/or Shark to DRPR. More recently, in mouse macrophages, the TRPV2 channel was shown to play a crucial role in particle recognition and phagocytosis by macrophages, where it promotes immunoreceptor clustering by inducing membrane depolarization and phospholipid-dependent actin depolymerization [59]. Thus PKD2 and/or TRPML may play similar roles in *Drosophila* macrophages and future studies will be necessary to determine the precise role of TRP channels in these cells in the context of apoptotic cells and bacteria clearance, as well as their precise role in calcium homeostasis during this process.

## Methods

### Fly strains

All fly stocks were obtained from the Szeged or Bloomington *Drosophila* stock centres, unless otherwise specified. Whenever possible, fly strains were crossed to a balancer chromosome containing a *kruppel::GFP (kr-GFP)* or *twist::GFP* (*twi-GFP*) reporter transgene and homozygous mutant embryos were selected against *kr-GFP* expression on a Leica fluorescent stereo-microscope equipped with GFP filter (Leica Microsystems Inc, UK), or detected by their lack of GFP immunoreactivity by confocal microscopy (Bio-Rad Radiance confocal equipped with a Nikon upright microscope). *crq-Gal4, UAS-eGFP* transgenic flies were a generous gift from Hervé Agaisse (Yale University) and Norbert Perrimon (Harvard Medical School). We generated *UAS-* transgenic flies by injecting *yw* embryos with a mixture of *UAS-*construct and helper plasmid at the concentration of 200 ng and 100 ng/µl respectively following standard procedures. Fly stocks were all maintained at 25°C in LMS600 incubators with 12 hour-light cycles (unless otherwise stated) on standard fly medium.

### Acridine orange (AO)

Embryos were collected for two hours at 25°C on juice plates and aged for 17 hrs at 18°C to stage 13. Embryos were treated with 50% bleach to remove their chorion, and washed extensively in bi-distilled water. Acridine orange (AO) staining was performed as described previously by shaking the embryo for seven minutes in an equal mixture of heptane and PBS containing 5 µg/ml of AO [34]. Stained embryos were transferred onto a glass slide, covered with halocarbon oil 700 and a coverslip, and subsequently viewed within 20 minutes under a Leica fluorescent MZ FL III stereo-microscope equipped with both green and red filters (Leica Microsystems Inc, UK).

### Immunostainings

Appropriately staged embryos were fixed in 4% formaldehyde/PBS (electron microscopy grade, TAAB), devitellinized with a 1∶1 volume mixture of methanol/n-Heptane, re-hydrated through a series of ethanol/PBT (25%/75%, 50%/50% and 25%/75%, 100%), blocked for 1 hr at room temperature in PBT containing 10% bovine serum albumin (BSA), and stained with either the CRQ Ab (IgG purified rabbit polyclonal antibody) alone, as previously described in [28] or using both the anti-CRQ and anti-GFP antibodies (mouse monoclonal antibody, Gibco) at 1∶1,000 and 1∶4,000 dilutions respectively in PBT containing 1% BSA at 4°C overnight. Three washes of 30 minutes in PBT were performed at room temperature and followed by 1 hr of incubation in PBT containing 1%BSA with appropriate fluorescent secondary antibodies. Anti-rabbit and anti-mouse fluorescein-coupled secondary antibodies were from Vector laboratories and used at a 1∶1,000 dilution. The anti-rabbit Cy5 antibody (Jackson Laboratories) was used at a 1∶1,000 dilution. After three washes for 30 minutes in PBT and two quick washes in PBS, a 7-amino actinomycin D (7-AAD) staining was performed in PBS at a concentration of 5 mg/ml, followed by three washes in PBS for 5–10 minutes [29]. Stained embryos were mounted in vectashield medium (Vector laboratories). Embryos were observed on a Bio-Rad Radiance confocal microscope equipped with a Nikon upright microscope using 25×, 40×, 60×, and/or 100× objectives. Images were collected using the lasersharp 2000 software and further processed using versions 4.0 or 6.0 of Adobe Photoshop software or Image J 1.34 g (NIH). For the VASA antibody staining, the VASA primary antibody made in chicken was diluted at 1∶10,000 in PBT, followed by detection with an anti-chicken secondary antibody coupled to FITC diluted at 1∶1,000 in PBT.

### Terminal deoxyribonucleotide transferase (TdT)-mediated dUTP Nick End Labelling (TUNEL)

Embryos were collected, staged, fixed and rehydrated as described above for immunostaining. Once in PBT, the embryos were treated with 10 µg/ml of Proteinase K in PBS for 4 minutes, followed by two washes of 5 minutes in PBT. The embryos were post-fixed in 4% paraformaldehyde (EM grade) in PBS for 20 minutes at room temperature on a rotating platform. After five washes for 5 minutes in PBT, the embryos were incubated for 1 hour at room temperature in equilibration buffer of the apoptag kit (Chemicon), followed by an overnight incubation at 37°C in 110 µl of reaction buffer containing 2 volumes of TdT and 1 volume of 0.3% Trito-X100, while shaking. Embryos are then incubated in 1 ml of Stop buffer for 3 hours at 37°C, while shaking, washed three times in PBT for 5 minutes, blocked in PBT containing 5% of Normal Goat Serum (NGS) and 2 mg/ml of BSA for 1 hour at room temperature. The incorporation of digoxygenin dUTP into the nicked-end of apoptotic cells' DNA is then detected using a FITC-coupled digoxygenin primary antibody (Roche) used at a 1∶100 dilution in fresh blocking buffer for 1 hour at room temperature. After three washes in PBT of 20 minutes each, the embryos are mounted between slides and coverslips in vectashield mounting medium (Vector), and observed using a Leica SP5 confocal microscope.

### Single embryo polymerase chain reactions (PCR)

Mapping of the deletions by PCRs were performed as previously described in [29] using template DNA extracted from homozygous embryos that were manually sorted against Kr-GFP expression. Primers used are listed in the **Supplementary Text S1**. In all reactions, a RP49 primer set was used as an internal control for the presence of DNA. RP49_S: 5′ ATACAGGCCCAAGATCGTGA 3′, RP49_AS: 5′ GTGTATTCCGACCACGTTACA 3′. PCR cycles were as follows: 94°C for 5 min for one cycle, denaturation at 94°C for 1 min, annealing at 65°C for 1 min, extension at 72°C for 1 min15 sec, for 30 cycles, and final extension at 72°C for 10 min. 15 µl of each PCR reaction were loaded onto a 2% agarose/TBE (Tris/Borate/EDTA) gel and their products were separated, visualized and photographed on a Syngene genelink trans-illuminating system.

### Plasmid construct

The *RE62284* EST containing the cDNA sequence of *Dmel\ced-12* (Berkeley Drosophila Genome Project (BDGP)) was obtained from the Berkeley *Drosophila* Genome Project (BDGP) collection library. The *Dmel\ced-12*cDNA insert was excised from the *pFLC1* vector following a triple restriction digests using 5 Units of the *KpnI*, *NotI*, and *BsphI* enzymes and appropriate buffer in the presence of 0.1 mg/ml of BSA. The KpnI/NotI cDNA insert was purified by gel extraction and ligated into the KpnI/NotI-digested *pUAST* vector using 5 Units of T4 DNA ligase in a 20 µl final volume overnight at room temperature. 1 µl of ligation was electroporated into *DH5alpha* bacteria from Invitrogen using a Bio-Rad electroporator and following manufacturer's instructions. Transformed bacteria were grown in 1 ml of SOC medium (Gibco) in a bacterial shaker at 37°C, and subsequently plated onto LB agar plates containing 100 µg/ml of ampicillin and incubated overnight at 37°C. Single colonies were picked and grown into 2 ml of LB medium containing 50 µg/ml of ampicillin. Preparations of plasmid DNA were performed using the Qiagen mini-prep kit. Individual clones containing the insert were identified by restriction map analysis and gel electrophoresis. Restriction digests were all conducted for one hour at 37°C and made use of 5 µl of each plasmid preparations and 5 Units of appropriate restriction enzymes in 20 µl final volume containing 10% volume of appropriate restriction buffer and 0.1 mg/ml of BSA.

### *In vivo* rescue experiment

To re-express *Dmel\ced-12*in macrophages in the *Df(2L)prd1.7* background, the following three stocks were established: (1) *y, w, UAS-dCed-12; Df(2L)prd1.7 /CyO*, (2) *w; Df(2L)prd1.7/ CyO; crq-Gal4, UAS-eGFP* and (3) *w, crq-gal4; Df(2L)prd1.7 /CyO*. The *crq-gal4* drivers were a kind gift of H. Agaisse and N. Perrimon, Harvard Medical School. The use of the *crq-gal4, UAS-eGFP* chromosome allows us to detect macrophages expressing cytoplasmic eGFP under the control of the *crq-gal4* driver. Females of the deficiency line carrying the *UAS-Dmel\ced-12* transgene were crossed to males of the deficiency line carrying the *crq-gal4* driver either on the X or on the third chromosome. Homozygous embryos were sorted for their segmentation defect on the confocal microscope prior to their analysis. All such embryos obtained with the *crq-gal4* on the third chromosome were rescued as they carried both one copy of the transgene and one copy of the driver. By contrast, when using the *crq-gal4* driver on the X chromosome, only half of the homozygous deficient embryos (the females) were carrying both the driver and the transgene on the X chromosome, as expected by Mendel's law. The fly line carrying the genomic rescue transgene of *prd* in the background of the *Df(2L)prd1.7* deficiency was a kind gift of Drs. Eric Frei and Marcus Noll (Institute for Molecular Biology, University of Zurich).

### Generation of Germ Line Clones (GLCs)

The *dced-12[19F3]*, *dced-12[PB]* and *dced-12[8c6]* mutations were recombined onto the FLP recombination target (FRT)-containing second chromosome of *w; a[1] dp ov[1] b[1] pr[1] P{ry[+t7.2] = neoFRT}40A* (Bloomington Stock Center) and crossed to males of a *hs-FLP, P{ry[+t7.2 = neoFRT}40A, Ubi-GFP/CyO* stock. The progeny from this cross was heat-shocked at 37C for 1 hour, three times with 24 hours of recovery between each heat-shock, starting at late L2 or L3 stages (i.e. on days 4, 5 and 6 of culture). Virgin females *hs-FLP, P{ry[+t7.2 = neoFRT}40A, Ubi-GFP/dced-12[19F3]*, or */dced-12[PB]*, or /*dced-12[8c6]* were recovered and crossed to heterozygous males *Df(2L)prd1.7/CyO, kr-GFP or Df(2L)prl/CyO, kr-GFP*. The phagocytosis phenotype of embryos resulting from this cross and sorted against GFP expression prior to fixation was assessed as described above. Of note is that no viable stage 13 embryos could be recovered when using the *dced-12[19F3]* allele.

### Statistical analyses

Phagocytic indexes (PIs) presented in this study are average PIs calculated from confocal images taken on five to 15 different embryos per genotype, in which confocal image-stacks of 12 to 24 sections through macrophages per embryo were taken at 60× and a zoom of 1.7. Standard errors of the mean (SEMs) were derived from the PIs calculated from all embryos per genotype. One way-analyses of variance (one way-ANOVAs) were performed to compare PIs between embryos of various genotypes that returned p values, which can be found throughout the text.

## Supporting Information

Figure S1 **Characterization of macrophage migration phenotypes in** ***deficient*** **and** ***prd^8^*** **homozygous mutant embryos.** In **A–F** embryos were aged to stage 13, fixed, their macrophages immunostained with the CRQ Ab (green) and apoptotic corpses detected with 7-AAD staining (red). Confocal images of twelve focal plans taken through the head of a wild-type *y,w* embryo (**A** and **D**), *Df(2L)prd1.7* (**B**) and *Df(2L)Prl* (**C**), *Df(2L)Esc-P3-0* (**E**) and *prd^8^* (**F**) homozygous mutant embryos. In all embryos, macrophages properly migrate throughout the head of the embryo surrounding the brain lobes, and appear large as they engulf multiple apoptotic cells. Scale bars are 50 µm.
(TIF)

Figure S2 **Characterization of macrophage phagocytosis phenotypes in** ***prd^8^*** **homozygous mutant embryos.** In **A–B** embryos were aged to stage 13, fixed, their macrophages immunostained with the CRQ Ab (green) and apoptotic corpses detected with 7-AAD staining (red). Confocal images of twelve focal plans were taken through the head of a wild-type *y,w* embryo (**A**) and *prd^8^* (**B**) homozygous mutant embryos. Macrophages in *prd^8^* appear larger as they engulf more apoptotic cells compared to wild-type macrophages. Scale bars are 10 µm. In A–B, dotted white circles are indicative of individual macrophage cell bodies based on 7-AAD staining of their regular nuclei and CRQ staining.
(TIF)

Text S1 **Additional Material.** This file lists the primers used to map the breakpoints of the deficiencies used in the present study (see **Material and Methods**).
(DOC)
