# Correction: The *Drosophila* TRPP Cation Channel, PKD2 and Dmel/Ced-12 Act in Genetically Distinct Pathways during Apoptotic Cell Clearance

**DOI:** 10.1371/annotation/8101d38a-2e75-480d-8d1c-016ba5360d5d

**Published:** 2012-04-26

**Authors:** Emeline Van Goethem, Elizabeth A. Silva, Hui Xiao, Nathalie C. Franc

The authors would like to make the following addition to the Acknowledgments section of this article: "The authors also wish to thank Christina Bakatselou for performing and providing images of the acridine orange stainings of Figure 1." 

