# Supplementary figures and images for: The Drosophila TRPP Cation Channel, PKD2 and Dmel/Ced-12 Act in Genetically Distinct Pathways during Apoptotic Cell Clearance
Source: PLoS One. 2012 Feb 8;7(2):e31488. doi: 10.1371/journal.pone.0031488 (PMC3275576; doi:10.1371/journal.pone.0031488)

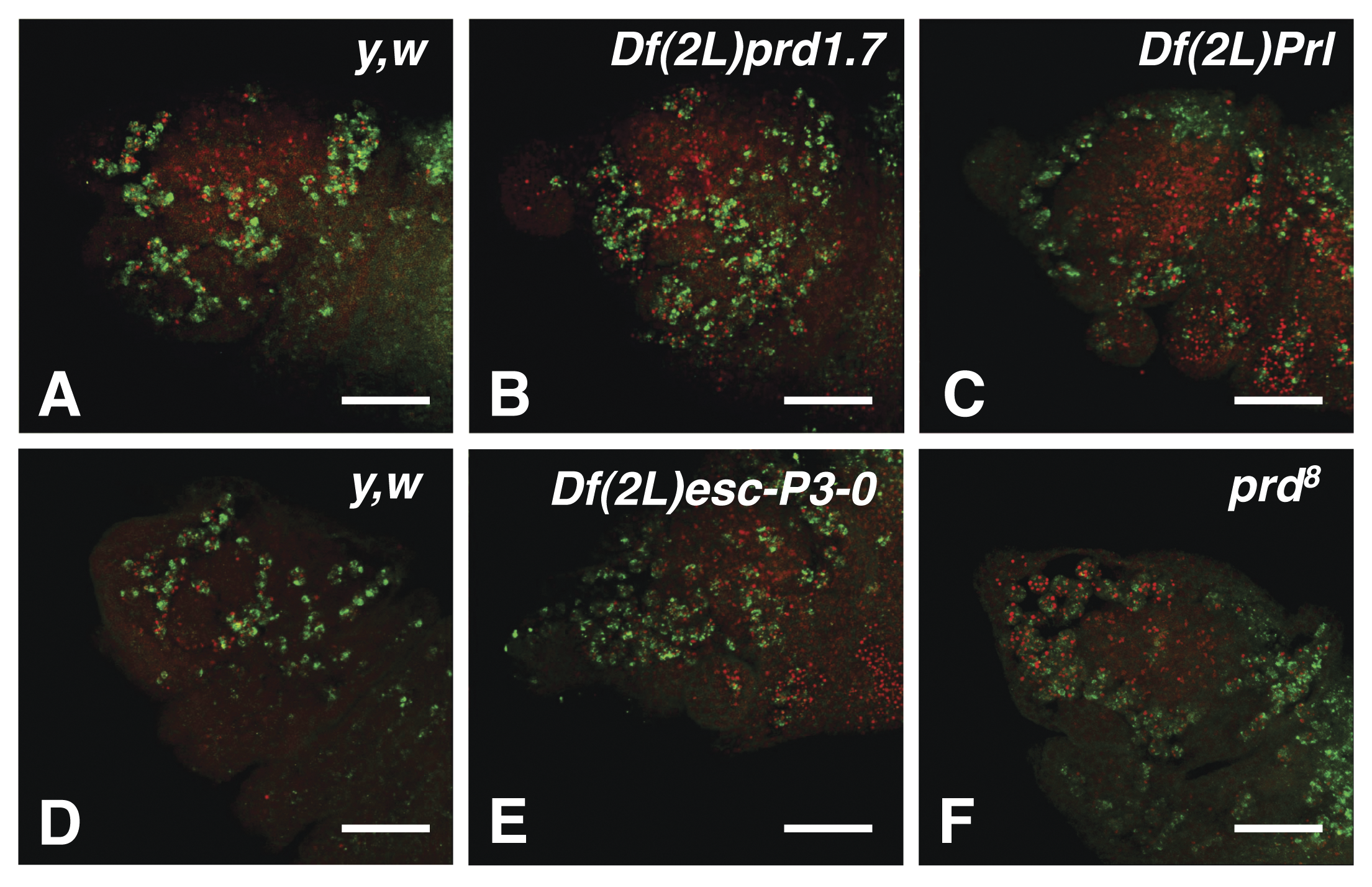

Supplement: Figure S1 — Characterization of macrophage migration phenotypes in deficient and prd8 homozygous mutant embryos. In A–F embryos were aged to stage 13, fixed, their macrophages immunostained with the CRQ Ab (green) and apoptotic corpses detected with 7-AAD staining (red). Confocal images of twelve focal plans taken through the head of a wild-type y,w embryo (A and D), Df(2L)prd1.7 (B) and Df(2L)Prl (C), Df(2L)Esc-P3-0 (E) and prd8 (F) homozygous mutant embryos. In all embryos, macrophages properly migrate throughout the head of the embryo surrounding the brain lobes, and appear large as they engulf multiple apoptotic cells. Scale bars are 50 µm. (TIF) [file pone.0031488.s001.tif]

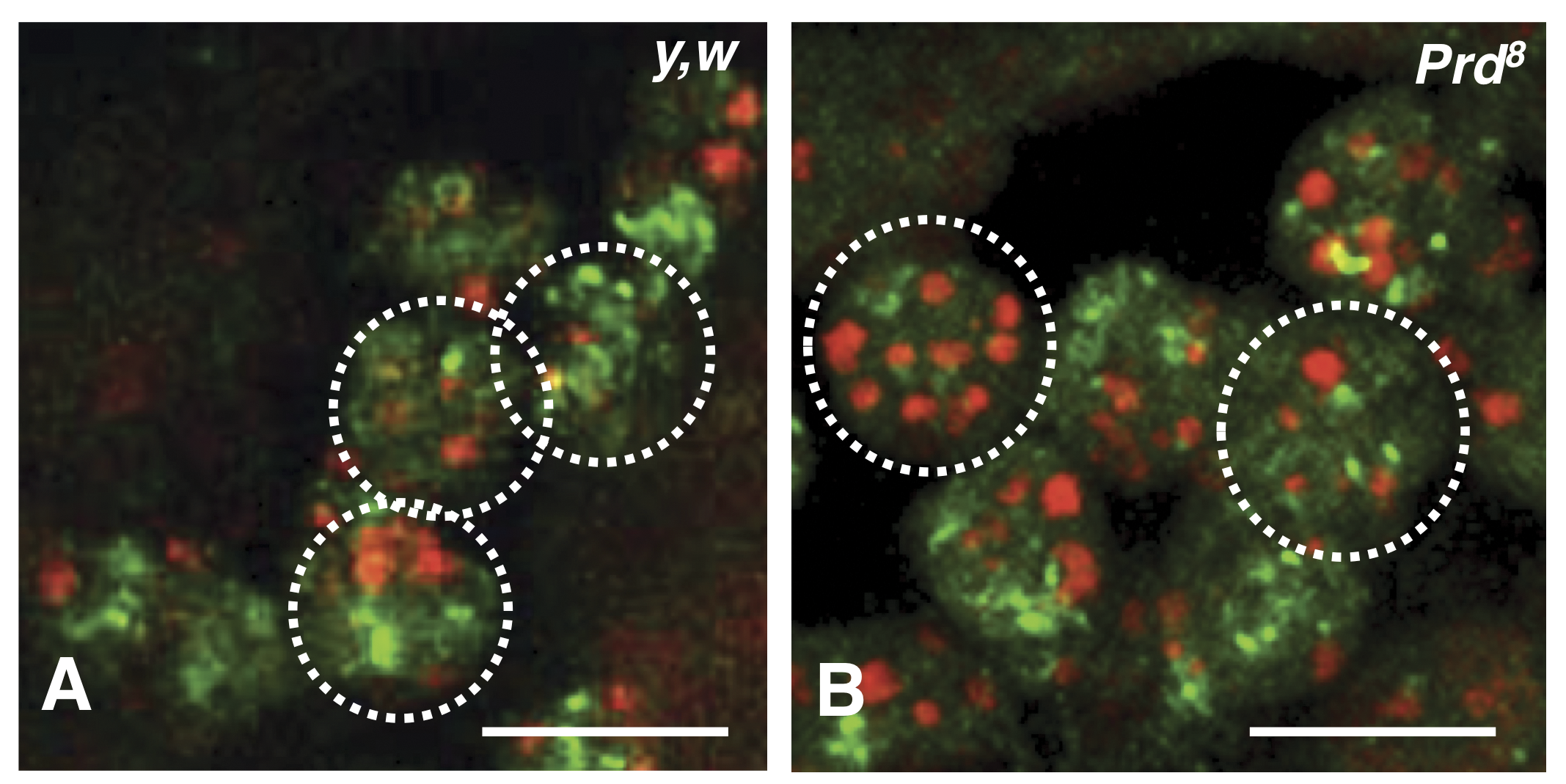

Supplement: Figure S2 — Characterization of macrophage phagocytosis phenotypes in prd8 homozygous mutant embryos. In A–B embryos were aged to stage 13, fixed, their macrophages immunostained with the CRQ Ab (green) and apoptotic corpses detected with 7-AAD staining (red). Confocal images of twelve focal plans were taken through the head of a wild-type y,w embryo (A) and prd8 (B) homozygous mutant embryos. Macrophages in prd8 appear larger as they engulf more apoptotic cells compared to wild-type macrophages. Scale bars are 10 µm. In A–B, dotted white circles are indicative of individual macrophage cell bodies based on 7-AAD staining of their regular nuclei and CRQ staining. (TIF) [file pone.0031488.s002.tif]
