# Supplementary material for: The Drosophila TRPP Cation Channel, PKD2 and Dmel/Ced-12 Act in Genetically Distinct Pathways during Apoptotic Cell Clearance
Source: PLoS One. 2012 Feb 8;7(2):e31488. doi: 10.1371/journal.pone.0031488 (PMC3275576; doi:10.1371/journal.pone.0031488)
Supplement: Text S1 — Additional Material. This file lists the primers used to map the breakpoints of the deficiencies used in the present study (see Material and Methods ). (DOC) [file pone.0031488.s003.doc]

# Supplementary text S1

### Supplementary material

Mapping primer sets

Map1-for 5’ TATTATCCCAGGGCATCCAA 3’;

Map1-rev 5’ DATTACACCTGCCGGAAAAA 3’;

Map3-for 5’ TGGCTTCTGCTGTAGGTGTG 3’;

Map3-rev 5’ CGCAAGCAAAAATTGCATAA 3;

Map5-for 5’ CATTAATCCCCCGAGCAATA 3’;

Map5-rev 5’ GGAACGTTCAAGATGGCACT 3’

Map6-for 5’ CCACACTTCCACTTCGGTTT 3’

Map6-rev 5’ AAGCGGAAACCCTTTTTGTT 3’;

Map7-for 5’ ACAGACGCTAGCTCCCGATA 3’;

Map7-rev 5’ CTGTCGGTGAAGGAAAGCTC 3’;

Map8-for 5’ AACTCGACTCGGTAGCAGGA 3’;

Map8-rev 5’ GACCGTGGATGAACAGGTCT 3’;

Map9-for 5’ AATCGGCAACCACTTTGTTC 3’;

Map9-rev 5’ GTTGCTGCCGTTGTTGTAGA 3’;

Map10-for 5’ TCAGCATCATCGTCAAGCTC 3’;

Map10-rev 5’ CCAGCGACAGTCAGTGAAAA 3’;

Map11-for 5’ CCCAAAAGGAAGTTGTGGAA 3’

Map11-rev 5’ CCAAAACTTTGCCCAACACT 3’;

Map12-for 5’ AGCCACCGAGTGATGTTACC 3’;

Map12-rev 5’ CGGGCAGTTCAAAACAAAAT 3’;

Map13-for 5’ CACCCAGCTAAGCGAGAATC 3’;

Map13-rev 5’ CATCCTGAGCAACTCGACAA 3’;

Map14-for 5’ AATGCCATACCGAGTCAAGC 3’;

Map14-rev 5’ CGCGAACGAATGACAGACTA 3’;

Map15-for 5’ AACGGTGGTTCTTCAACCTG 3’;

Map15-rev 5’ GACGCTTATTTTTCGCTTCG 3’;

Map16-for 5’ ACGCCGTGCTGAAAAATAGT 3’;

Map16-rev 5’ CCACAAGGGGAGGTCAGTTA 3’;

Map17-for 5’ TGCCTCGTTGATTAGCACAG 3’;

Map17-rev 5’ ATAAGCAGCCAGAGGGGAAT 3’;

Map18-for 5’ TTTCCCAGGCATTTCTCATC 3’;

Map18-rev 5’ ACCACGATCCACTTCCACTC 3’;

Map19-for 5’ ATCGAGGAGCTTGGCTTGTA 3’;

Map19-rev 5’ CCAAATGGCAGGAATCTCAT 3’;

Map20-for 5’ CACATTCGCCACTAAGCAGA 3’;

Map20-rev 5’ CGCTAGTGCGCTAGTGTGAG 3’;

Map21-for 5’ GTTTAGGCCACACAGCCATT 3’;

Map21-rev 5’ CTCGAAGTTCACCTTGCACA 3’;

Map22-for 5’ CCCGTAAACGTTTGCTGATT 3’;

Map22-rev 5’ GTGTGGTATGTGTGCGAAGG 3’;

Map23-for 5’ GCAAGCAGATCGAGGGTAAG 3’;

Map23-rev 5’ GGTTGATTTTTGCGTTTCGT 3’;

Map24-for 5’ AGCAGGGTCAGAGTGGAAGA 3’;

Map24-rev 5’ CCGAACGGACAAAACAACTT 3’;

Map27-for 5’ CAACCGGTACTCGTCGATTT 3’;

Map27-rev 5’ TGTTCAAACCGCAATCGTAA 3’;

Map28-for 5’ GGTGCACAAAACAAATGCAC 3’;

Map28-rev 5’ TTGGCTTTCCCAAAAATCTG 3’;

Map29-for 5’ CGCCAACTTATCCCACAACT 3’;

Map29-rev 5’ ATGTGGCGACGATATGACAA 3’;

Map30-for 5’ GCAAACCGAACAAGAGCTTC 3’;

Map30-rev 5’ GCGACTGAAACATTCAGCAA 3’;

Map31-for 5’ CAATCTTCGACTGGCTGTCA 3’;

Map31-rev 5’ CAGATTCCTCGTCCTCTTCG 3’;

Map32-for 5’ GGCTCTGGAAAAGAGCACAC 3’;

Map32-rev 5’ CGTAGCAGCTTCCTGTTTCC 3’;

Map33-for 5’ ACACCAGCACAGAACAGCAC 3’;

Map33-rev 5’ CGTGAACTCAGCATCTTCCA 3’;

Map34-for 5’ GGTTGCCTTTTTGACGACAT 3’;

Map34-rev 5’ CAGACGTTCAGTCGCCAGTA 3’;

Map35-for 5’ CCAATTGCCGCCTTTATTTA 3’;

Map35-rev 5’ CCAGGAGAAAGCAGAGAACG 3’;

Map36-for 5’ AAAAAGATCGCTCTCGCAAA 3’;

Map36-rev 5’ TCCTTGTGGAGAAACCAACC 3’;

Map37-for 5’ TGTGGATTTCAGGTCAACCA 3’;

Map37-rev 5’ CAAACAGCAAATCGCAGAAA 3’;

Map38-for 5’ TTGGTTCGCTTCTTTGCTTT 3’;

Map38-rev 5’ CGAAGTGGTCAGTTGCGATA 3’;

Map39-for 5’ CAGGGCAGACAGAACTCACA 3’;

Map39-rev 5’ GGTTAGCCAGAAGCTCGTTG 3’;

Map40-for 5’ ACCCCAGCACTTTGCATATC 3’;

Map40-rev 5’ AGTGAAAAACCGGTGCAAAC 3’;

Map41-for 5’ TCGGCTTGTCCTCAGAAGTT 3’;

Map41-rev 5’ ATATTTCCCCCTGAGGGTTG 3’;

Map42-for 5’ TCCAGCTGCGAGGAAGTAAT 3’;

Map42-rev 5’ CAAAGTTGGGCAGTTGGTTT 3’;

Map43-for 5’ AAAGCAAAGGCGCAGATAAA 3’;

Map43-rev 5’ GCCTTTCTGTGCTTCACTCC 3’;

Map44-for 5’ GAGCTTTCCCAAGTGTCTGC 3’;

Map44-rev 5’ ATATCGGGGCTTTCGCTATT 3’;

Map45-for 5’ ACCGAATGAAAACCAAGTCG 3’;

Map45-rev 5’ GTGGTTGGGTAATGGTTTGG 3’;

Map46-for 5’ CTTGAGCTTTTTGGGCTTTG 3’;

Map46-rev 5’ ATGGACCGATATTTGGGTGA 3’;

Map47-for 5’ GCTCGCTGTTGGCTATTTTC 3’;

Map47-rev 5’ AATTAAAACCCCCTGCCATC 3’;

Map48-for 5’ GCATTTCAGCGAAACTGTCA 3’;

Map48-rev 5’ AACATGTGCAGACCCATTGA 3’;

Map49-for 5’ AATGCCGTCAGTGGTAGTCC 3’;

Map49-rev 5’ GAGCAGGCGATCGTAAAGAG 3’;

Map50-for 5’ TACCGGCGAAAGGTGTTTAC 3’;

Map50-rev 5’ GCTCAGCTTTTCGTTTCCAC 3’;

Map51-for 5’ CGACCATTCGCCTAATGAAT 3’;

Map51-rev 5’ GTCTGCTCACGTCCTTGTC 3’;

Map52-for 5’ TGGAGAATGTGCGTCTTGAG 3’;

Map52-rev 5’ AGTCGTCCCAGAAGCAGAAA 3’;

Map53-for 5’ AAACCACAAAAGCGAACCAG 3’;

Map53-rev 5’ ACGCAAACCGAAAATGTAGG 3’;

Map54-for 5’ AATTTGTTGCTGCTGCCTCT 3’;

Map54-rev 5’ GGCACGTCCGTTTCTTGTAT 3’;

Map55-for 5’ CGTTTTGCGTCTGAACAAGA 3’;

Map55-rev 5’ GGTGAGCAGACAGATGACGA 3’;

Map56-for 5’ AAACGAACAACGACCCTTTG 3’;

Map56-rev 5’ CCGAAGATTCCGAAAAATCA 3’.
